# Meningeal Tertiary Lymphoid Tissues and Multiple Sclerosis: A Gathering Place for Diverse Types of Immune Cells during CNS Autoimmunity

**DOI:** 10.3389/fimmu.2015.00657

**Published:** 2016-01-13

**Authors:** Natalia B. Pikor, Alexandre Prat, Amit Bar-Or, Jennifer L. Gommerman

**Affiliations:** ^1^Department of Immunology, University of Toronto, Toronto, ON, Canada; ^2^Neuroimmunology Unit, Department of Neuroscience, Centre de Recherche de CHUM, Université de Montréal, Montreal, QC, Canada; ^3^Neuroimmunology Unit, Montreal Neurological Institute and Hospital, McGill University, Montreal, QC, Canada

**Keywords:** meninges, multiple sclerosis, experimental autoimmune encephalomyelitis, tertiary lymphoid tissues, stromal cells

## Abstract

Collections of leukocytes in the meningeal space have been documented in Multiple Sclerosis (MS). These meningeal aggregates, which in the context of other autoimmune diseases have often been termed tertiary lymphoid tissues (TLT), have been associated with sub-pial cortical damage and disease progression. However, the key molecular and cellular signals required for their formation and maintenance remain unclear. Herein, we review TLT structures in other disease states in order to provide a framework for understanding these structures in the MS meninges. We then assess the evidence that the meningeal compartment serves as an important nexus for immune cells as well as a location for drainage of antigen into cervical lymph nodes. Extrapolating what is known about the molecular and cellular cues that initiate the formation of leukocyte aggregates in non-lymphoid tissues, we speculate on what signals lead to the formation and maintenance of meningeal TLT structures. Referring to the animal model of MS [experimental autoimmune encephalomyelitis (EAE)], we also explore what is known about these structures in supporting B cell and T cell responses during neuroinflammation. Last, we examine the evidence that connects these structures to ongoing neuropathology. Collectively, our review points to the meningeal compartment as an important player in neuroinflammatory processes. Moreover, we hypothesize that in order to gain insights into pro- and anti-inflammatory properties of lymphocytes in MS, one must understand the cellular scaffolds that support lymphocyte retention within the meninges, thus highlighting the importance of non-immune cells (stromal cells) in the neuroinflammatory process.

## Overview

Multiple sclerosis (MS) is an inflammatory disease of the central nervous system (CNS) resulting in demyelination and axonal loss with consequential clinical impairment. MS is most commonly diagnosed as relapsing-remitting MS (RRMS), which is thought to reflect the waxing and waning of underlying CNS-targeted immune responses. Most individuals with RRMS go on to develop a progressive form of MS, termed secondary progressive MS. The relative absence of new gadolinium-enhancing lesions in the CNS of individuals with progressive MS has for a long-time supported the hypothesis that there is limited infiltration of peripheral immune cells into the CNS at this stage of disease (1). This concept has been recently challenged following the observation of meningeal leukocytic infiltrates (consisting of T cells, B cells, plasma cells, monocytes and macrophages) in both SPMS [in as many as 40% of cases; (2–4)] and Primary Progressive MS (5), correlating with proximal neuropathology (6). However, meningeal inflammation may also contribute to neuroinflammatory processes early on in MS, and recent MRI studies demonstrate leptomeningeal contrast enhancement in RRMS [~19% of cases; (7)].

Meningeal leukocytic aggregates have been referred to as tertiary lymphoid tissues (TLT), provoking the hypothesis that such aggregates support disease-relevant immune responses locally within the CNS. Although there is some discrepancy as to whether meningeal follicle-like structures recapitulate all of the features of TLT, for the purposes of this review, we will refer to them as TLT (8–10).

## Tertiary Lymphoid Tissues

Tertiary lymphoid tissues (TLT) are locally inducible leukocyte aggregates that form in chronically inflamed non-lymphoid tissues and share cellular and organizational similarities with secondary lymphoid organs (SLO). TLT arise within the target tissues of many autoimmune diseases and certain sites of chronic infection, including in the synovial membrane of the joints (rheumatoid arthritis), salivary glands (Sjögren’s syndrome), thymus (Mysasthenia gravis and Grave disease), meninges (MS), the liver (hepatitis C viral infection), the lung (Influenza A viral infection), as well as at sites of chronic graft rejection, atherosclerosis, and cancer (8, 10). The majority of information we have on TLT structure and formation is from disease settings that do not involve the CNS. This may be due to the limitations in studying TLT in post-mortem tissue (brain tissue from autopsies tends to be obtained very late in the disease process when inflammation may be less pronounced), the relatively smaller size of meningeal TLT, and variability in histology and dissection protocols for assessing meningeal TLT. As such, this section will focus on what we have learned about TLT in other disease settings that may be more amenable to study, and we will then apply these findings to CNS autoimmunity in a subsequent section.

### TLT Structure

Lymphoid architecture is orchestrated by specialized stromal cell subsets. Follicular dendritic cells (FDC; CD45^−^CD31^−^Pdpn^±^) and recently defined *Cxcl12*-expressing reticular cells (CRC; CD45^−^CD31^−^Pdpn^±^) (11) secrete B cell chemoattractants CXCL13 and CXCL12, respectively. FDC further upregulate molecules involved in trapping and presenting antigen (CD35, FDC-M1, and FDC-M2) to support germinal center responses. The T cell zone is supported by fibroblastic reticular cells (FRC; CD45^−^Pdpn^+^CD31^−^) that secrete T cell chemoattractants (CCL19, CCL21) as well as T and B cell survival factors (IL-7, BAFF) and form long reticular channels supporting the passage of antigen through the lymphoid organ (12). TLT encompass a spectrum of lymphoid tissue-like organization depending on the target tissue. TLT are primarily described as B cell-rich infiltrates, with a varying degree of T cell infiltration and sometimes segregation into distinct B and T cell compartments resembling SLO architecture. Although FDC markers have been detected within TLT in various chronic inflammatory conditions, not all TLT demonstrate germinal centers or reticular conduits reminiscent of FRC (10, 13, 14). Mature TLT can also contain high endothelial venules (HEV) and lymphatic vessels, suggesting an avenue for entry of naive lymphocytes (via the HEV) and egress of antigen and activated or memory lymphocytes via lymphatic vessels (15).

### TLT Formation

Lymphorganogenesis requires the well-defined interaction of embryonic/neonatal lymphoid tissue inducer cells (LTi; CD45^+^CD4^+^CD3^−^) with stromal lymphoid tissue organizer cells (LTo) to promote LTo maturation into specialized lymphoid stromal cells that in turn form an immune-competent niche [reviewed by Ref. (12, 16–20)]. In the context of lymphoneogenesis in the adult host, distinct cell types may substitute as TLT inducer and organizer cells. LTi equivalent cell types implicated in TLT formation include: innate lymphoid cells (21–23), T cells (24–27), NKT cells (28), as well a myeloid cells (28, 29). Although the exact combination of molecular cues may differ, a unifying feature of TLT-inducing leukocytes is their production of cytokines, especially IL-17 [reviewed by Ref. (30)] and/or IL-22 (21, 22, 27), and their ability to engage receptors of the Tumor Necrosis Factor (TNF) superfamily (LTβR, TNFR) by virtue of their expression of cognate ligands LTαβ and TNFα.

The origin and phenotype of TLT organizer cells remains more elusive. Seminal studies have demonstrated that stromal precursor cells reside quiescently throughout the periphery, and mature to acquire phenotypic and functional capacities consistent with lymphoid tissue stromal cells in response to inflammation (29, 31). A population of CD45^−^CD31^−^Pdpn^−^ cells, which transcriptionally most closely resemble FRCs (32), has also been demonstrated to differentiate into *de novo* FRCs in the inflamed LN (33, 34), illustrating that even within adult SLOs, mesenchymal precursors can recapitulate an LTo-like function. Nevertheless, further studies are needed in order to elucidate to what extent mesenchymal precursor cells in the adult host resemble embryonic LTos and how such LTo-like cells differentiate in order to support emerging TLT.

Several markers have been useful for assessing the phenotype of tissue-resident stromal cells, including: podoplanin (Pdpn or gp38); the endothelial cell marker, CD31; EPCAM, a marker of epithelial cells; as well as the expression of homeostatic chemokines. For example, in several models of TLT formation, peripheral Pdpn^+^ stromal cells express CXCL13 (26, 27, 29, 31), while in a model of atherosclerosis, vascular smooth muscle cells were found to express both CXCL13 and CCL21 within aortic TLT (35). Both Pdpn^+^CD31^−^ stromal cells and EPCAM^+^ epithelial cells express CXCL12 within TLT in the lungs (26) and salivary glands (27), respectively. Nevertheless, without a consistent panel of mesenchymal/lymphoid stromal cell markers applied to different models of TLT formation, it is difficult to interpret whether different inflammatory insults instigate distinct maturation protocols from ubiquitous precursor cells, or whether tissue-specific differences exist.

In summary, our current understanding is that precursors of lymphoid-like stromal cells reside quiescently throughout peripheral non-lymphoid tissues and are poised to mature into lymphoid stromal cells at sites of persistent inflammation. In response to local inflammatory cues, tissue-resident stromal cells acquire phenotypic and functional capacities consistent with lymphoid tissue stromal cells.

## Structure and Function of Meningeal TLT during CNS Autoimmunity

Meningeal aggregates in the MS CNS are often referred to as TLT; however, as is true for ectopic lymphoid tissues in general, this term captures a range of lymphoid tissue-like organization. Animal studies characterizing meningeal inflammation in EAE demonstrate TLT formation in mice with different genetic backgrounds and disease-induction protocols (24, 36–39). These meningeal infiltrates often resemble mature TLT, with the presence of lymphoid-like stromal cells, elaboration of an extra-cellular matrix (ECM) network, and expression of cytokines and homeostatic chemokines. Below, we will review our current understanding about the structure and capacity for the meninges to support TLT formation, as well as the clinical and neuropathological correlates of meningeal TLT in both MS and EAE (see also Table 1).

**Table 1:** **Association of immune cell phenotypes and pathology with TLT**.

Feature	Evidence	Reference
B cell responses – EAE	B cell-rich meningeal aggregates during EAE	(36)
		(40)
		(24)
	FDC-M1- and CD35-immunoreactive cells (FDC-like cells) and CXCL13 transcripts within meningeal TLT	(36)
B cell responses – MS	B cell- and plasma cell-rich meningeal TLT	(2)
		(41)
	CD35- and CXCL13-immunoreactive cells (FDC-like cells) within meningeal TLT	(2)
	Activated B cells (clonal expansion, somatic hypermutation, Ig class switching) within meningeal aggregates	(42)
		(43)
		(44)
		(45)
T cell responses – EAE	T cells infiltrate the meninges and are reactivated in the subarachnoid compartment	(46)
		(47)
		(48)
		(49)
		(50)
	T cell epitope spreading concurrent with presence of meningeal TLT	(37)
	Th17 cells contribute to meningeal TLT formation	(51)
		(24)
		(52)
T cell responses – MS	T cell accumulation within meningeal TLT	(4)
		(53)
		(5)
Neuropathology – MS	Cortical demyelination	(54)
		(3)
		(6)
		(53)
		(4)
		(5)
	Glial limitans damage, increased microglial activation	(3)
		(6)
		(4)
		(41)
	Cortical astrocyte and oligodendrocyte loss	(6)
	Neuronal loss	(6)
		(5)
Clinical correlates	Earlier age of clinical onset, faster time of disease progression, earlier age at death	(3)
		(4)

### Anatomical Structure of the Meninges

**Figure 1:**
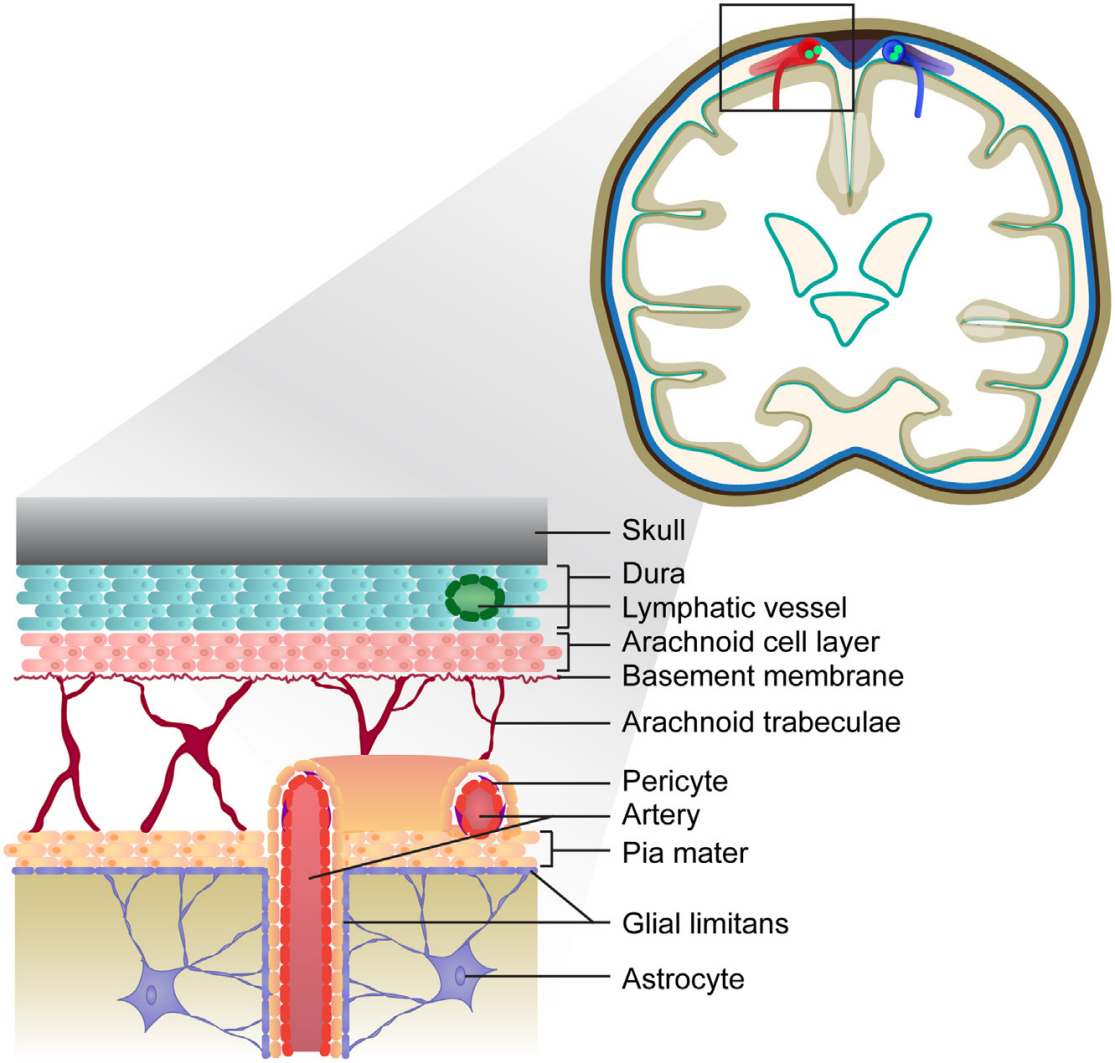
**Depiction of the meninges in the healthy brain**. The meninges consist of three layers: the dura is the outermost layer, followed by the arachnoid and pia, which form the leptomeninges. Lymphatic vessels embedded within the dura drain the sagittal sinus (not depicted). The vasculature transecting the meninges is embedded within the pial cell layer, and represents a route of leukocyte entry into the CNS.

The meninges are a series of membranes that envelope the brain and spinal cord, serving as a canal for circulating cerebrospinal fluid (CSF). The outermost membrane is the dura, which cocoons the CNS and is attached to the skull and spinal column. The leptomeninges that envelope the entire CNS consist of the arachnoid and pia mater and are separated by the subarachnoid space. Large conducting blood vessels transecting the leptomeninges are embedded within the pia mater, which is lined by the glial limitans, a barrier comprising astrocytic end-foot processes (55). Cells of the pia mater continue to line intracerebral arteries but gradually become less dense as the arteries penetrate the CNS parenchyma (56–58). The meningeal space is depicted in Figure 1. Cells that reside in the meningeal compartment include fibroblasts and peri-endothelial cells (myofibroblasts, pericytes, and vascular smooth muscle cells), as well as CNS-resident macrophages and dendritic cells. A recent study by Louveau and colleagues has revealed the presence of lymphatic vessels within the dural sinuses, implying there is direct communication between the meningeal environment and the draining cervical LNs (cLN) (59).

### The Meninges – A Portal of Leukocyte Entry and Accumulation in the Inflamed CNS

The blood endothelium transecting the subarachnoid space (the blood–CSF barrier) represents an important route of leukocyte entry into the meninges. In the steady state, the subarachnoid space represents an avenue for immune-surveying lymphocytes to scan the CNS (60). Studies examining the kinetics of meningeal infiltration in EAE report an influx of immune cells prior to clinical onset (39). In addition, using specialized fluorescent reporter mice and two-photon live imaging, myelin-specific T cells have been shown to first cross the blood–CSF barrier in the subarachnoid space, where antigen-specific re-priming must occur in order to gain access to the parenchyma and instigate clinical symptoms of EAE (47). With respect to MS, biopsies from early stage MS patients identified a subset of patients with evidence of cortical demyelinating lesions associated with meningeal inflammation (53). Moreover, a recent study has estimated that almost 20% of individuals with RRMS demonstrate meningeal contrast enhancement (7).

EAE studies, post-mortem histological analyses, and CSF samplings all demonstrate that accumulation of proliferating, antigen-experienced T cells (48, 61, 62), and B cells (3, 4, 42–45) can occur within the meningeal compartment itself during CNS autoimmunity. In terms of T cell responses, epitope spreading of myelin-reactive T cells is suggested to occur within the meninges (37). While antigen-presenting phagocytic cells have been shown to productively interact with myelin-specific T cells in the subarachnoid space (46, 48–50), recent studies also demonstrate that meningeal stromal cells may be important for propagating encephalitogenic T cell responses within the CNS (52). With respect to meningeal B cell responses, class-switched memory B cells and plasmablasts/plasma cells have long been detected in the CSF of individuals with MS, and contribute to intrathecal production of antibodies, a hallmark of MS (43, 63). While ­antigen-experienced B cells populate the meningeal compartment, clonally related B cells are also located in parenchymal lesions, the normal-appearing white matter (42), and in the periphery (44, 45, 64), making it unclear where B cells are first primed. The presence of myelin antigens in the cLN of individuals with MS, but not healthy controls (65), and the discovery of lymphatics draining from the meningeal space to cLN (59) suggest that antigen-dependent B cell responses can be initiated in the cLN. Recently, deep sequencing analyses of the B cell receptor variable heavy chain (VH) between matched CNS and peripheral samples demonstrated that upwards of 90% of founder B cell clones were localized within cervical lymph node tissues (44). However, it remains possible that the spatial–temporal distribution of founder clones differs at disease onset. In summary, the blood–CSF barrier is an important portal of entry for leukocytes into the CNS. The subarachnoid space represents an important site of accumulation for activated lymphocytes, as well as dendritic cells (46), neutrophils, and mast cells (39, 66) within the inflamed CNS.

### How Do TLT Form within the Meninges?

The presence of immune cells in the meninges and CSF of individuals with MS does not in and of itself confirm that the meninges constitute an immune-competent niche. To endow “immune competence,” such an environment would need to be populated by stromal support cells that secrete lymphocyte chemoattractants (such as CXCL13, CCL19, CCL21) and possibly also survival/differentiation factors (cytokines). Indeed, meningeal stromal cells have the capacity to secrete mediators such as TNFα, iNOS, IL-6, TGF-β, and IL-23 under inflammatory conditions (52, 67). Pro-inflammatory cytokines themselves can induce lymphocytic accumulation within the meningeal compartment, as demonstrated following injection of TNFα and IFNγ directly into the subarachnoid space (68) or intra-cortically (69) in rodents immunized sub-clinically with MOG. While these studies did not examine whether cytokine-induced TLT-like aggregates are supported by an underlying stromal cell and ECM network, TLT surrounded by a reticular network have been observed upon adoptive transfer of IL-17 producing encephalitogenic T cells in mice (24).

Expression of both T cell and B cell chemoattractants and lymphoid-like FRCs and FDCs has been demonstrated in the inflamed CNS. In both EAE and MS, CXCL13 expression and FDC-like cells (CD35^+^ and FDC-M1^+^) are reported within B cell-rich meningeal aggregates (2, 36, 40), while CCL19 and CCL21 transcripts have been detected within parenchymal and sub-meningeal lesions in the brains of SWR/J × SJL/J F1 mice with EAE (38). In the context of MS, over three decades ago, Prineas and colleagues described the presence of reticular-like cells embedded within lymphoid-like structures and lymphatic capillaries within old plaques in the MS CNS (70). While the phenotype of these reticular cells has not since been explored in the MS CNS, the elaboration and maturation of a reticular lymphoid-like stromal cell network were recently described in the brain meninges of mice with Th17 adoptive transfer EAE (52). FRC-like stromal cells were found to secrete pro-inflammatory cytokines, homeostatic chemokines (CXCL13, CCL21), as well as CXCL1 and BAFF, forming an immune-competent microenvironment. IL-17- and IL-22-derived signals were shown to promote the physical elaboration of the reticular network while acquisition of lymphoid-like stromal cell properties was in part lymphotoxin dependent, suggesting that multiple pathways culminate in the elaboration of an immune-regulatory stromal cell scaffold in the inflamed CNS. In summary, the meninges are a CNS environment poised to establish an inflammatory niche that is capable of supporting immune-responses within the CNS.

### Meningeal Inflammation and Cortical Pathology in MS

Cortical lesions characterized by demyelination, axonal atrophy, and microglial activation in the sub-pial mater have been shown to underly meningeal lymphocytic infiltrates in the progressive MS CNS (3, 5, 54), although another study failed to see a correlation between meningeal inflammation and sub-pial demyelination (71). The presence of meningeal TLT in SPMS correlates with accelerated clinical disease (earlier age of clinical onset, faster time of disease progression and earlier age of death) compared to SPMS cases without meningeal TLT (4). It is postulated that soluble factors emanating from meningeal lymphocytic aggregates degrade the glial limitans, promoting a gradient of demyelination and neuronal injury (3, 6). While B cells, plasma cells (IgA^+^, IgG^+^, IgM^+^), CD4^+^ and CD8^+^ T cells, monocytes/macrophages infiltrate the subarachnoid space, a recent study revealed that only the accumulation of plasma cells and macrophages was significantly elevated in meningeal TLT compared to region-matched controls, and the accumulation of these particular cell types was associated with underlying cerebellar gray matter demyelination (41). In addition, the accumulation of meningeal CD3^+^ T cells correlates with axonal loss and microglial activation in the underlying normal-appearing white matter in the spinal cord in progressive MS (72). Taken together, these observations may reflect distinct susceptibility of different regions of the CNS to immune cell-mediated injury.

## Conclusions

The presence and inducible formation of an immune-competent niche in the meninges suggests that these structures may support disease-relevant immune responses in the CNS. While TLT-associated immune responses are proposed to contribute to ongoing neuropathology and disease exacerbation, these structures likely evolve to support cell types regulating the balance of pro-inflammatory and anti-inflammatory responses in the meninges. Indeed, the accumulation of regulatory T cells within chronic aortic TLT is associated with clinical benefit in a rodent model of atherosclerosis (35). On the other hand, the presence of TLT in cases of enteropathic infection or cancer is associated with clinical benefit (21, 22, 73, 74). Therefore, one must consider that the cellular constituents of meningeal TLT may change over time, implicating altered neuropathological and clinical consequences within the inflamed CNS. We propose that an understanding of the cellular scaffolds that support lymphocyte retention within the meninges (i.e., specialized non-immune stromal cells) will lead to a better understanding of the meningeal compartment in the context of MS/EAE.

## Conflict of Interest Statement

Amit Bar-Or has participated as a speaker in meetings sponsored by and received consulting fees and/or grant support from Biogen Idec, Diogenix, Genentech, Sanofi-Genzyme, GlaxoSmithKline, Novartis, Ono Pharma, Teva Neuroscience, Receptos Inc., Roche, and Merck/EMD Serono. Jennifer Gommerman has received consulting fees from Novartis and Merck although these relationships bear no relevance to this mini-review. No other authors have financial relationships with entities that could be perceived to influence, or that give the appearance of potentially influencing, what the authors wrote in the submitted work.
